# The quality and scope of health information on online drug platforms: a topic modelling and expert evaluation study of a Polish-language forum

**DOI:** 10.1186/s12954-026-01424-y

**Published:** 2026-02-16

**Authors:** Piotr Siuda, Paweł Matuszewski

**Affiliations:** 1 Kazimierz Wielki University, Bydgoszcz, Poland; 2 Civitas University, Warsaw, Poland

**Keywords:** Health information quality, Consumer health information, Drugs, Psychoactive substances, Forums, Online platforms, Topic modelling, Natural language processing, Expert evaluation, Harm reduction

## Abstract

**Background:**

Determining health information quality on online drug platforms is crucial for revealing and shaping substance use practices. The study aims to assess the scope and quality of health-related information on the largest Polish drug forum, Hyperreal. Ultimately, the goal was to explore how the case of Hyperreal illustrates user-driven knowledge construction around drug use and its potential implications for harm reduction strategies on similar online platforms.

**Methods:**

A multimethod approach was employed for the current research. First, the full content of the forum was scraped, and topic modelling was used to pinpoint and analyse posts (*N* = 159,145) that discussed drugs in a health-related context. From this, 25 posts were selected for expert analysis using specified selection criteria for each topic. As existing standardized tools were found to be unsuitable for the aims of this study, the research team developed a custom questionnaire to evaluate the quality of posts, with psychiatrists serving as expert reviewers.

**Results:**

The topic distribution analysis indicated the most frequent and only sporadically discussed themes. Semantic analysis revealed distinct relationships between specific psychoactive substances and health topics, including associations between ketamine and depression/psychotherapy, marijuana and symptom relief and inflammation, amphetamines and cardiovascular issues, and fentanyl and palliative or oncological care. These patterns closely mirrored associations described in the medical literature, indicating knowledge structures grounded in medical rather than purely anecdotal knowledge. However, although the posts largely adhered to evidence-based medicine, the expert evaluation showed that they contained significant informational gaps. Overall, the mean quality of posts was *M* = 35.67, SD = 6.70, which indicates moderate health information quality. Experts rated factual accuracy and consistency with medical guidelines as moderate, while safety-related aspects—particularly dosage guidance and encouragement to consult specialists—received the lowest scores.

**Conclusions:**

Online drug platforms are an important source of knowledge on psychoactive substances. However, the health information quality of posts was average, which is especially crucial as these forums shape discourses that replace professional narratives. The study is relevant in the context of harm reduction strategies and emphasizes the need to improve the quality of drug platforms’ content.

**Supplementary Information:**

The online version contains supplementary material available at 10.1186/s12954-026-01424-y.

## Introduction

### Background

Online forums for drug users have recently received growing scholarly attention, particularly for their role in facilitating the sharing of drug use experiences and peer support [1–3]. In the process, these websites provide users with resources for constructing and reconstructing the narratives needed to maintain or change their sense of self-identity. Bilgrei [4] described these processes as *community consumerism*, a concept that aims to illuminate how online groups facilitate and empower alternative discourses regarding the impact of psychoactive substances on health. He emphasized the strong sense of togetherness in the discussions and described this involvement as crucial for building self-empowerment in health. According to Bilgrei [4], these platforms facilitate alternative discussions among users, including how they perceive the risks associated with drug use and which harm reduction strategies they adopt.

These strategies are usually well and spontaneously adopted by people who use drugs; that is, they are interested in mitigating the threats and unwanted consequences of drug use [3, 5, 6]. However, as mentioned above, research has indicated a clear shift away from professionalized, top-down narratives and toward peer-generated, bottom-up discourses that emphasize self-regulation and the benefits of drug use [7, 8]. The users find many “good motives” for using drugs, such as self-knowledge or self-enhancement [1].

For this reason, it seems crucial to research the quality of health information (also known as health information quality or HIQ) on drugs in various internet forums. Determining health information quality is not only important for judging the potential of these websites in relation to harm reduction but can also contribute to interventions that shape substance use practices and, therefore, influence the risks associated with drugs.

In the Polish context, drug use is shaped by relatively restrictive legal regulation and limited access to comprehensive drug education and harm reduction resources. Although illicit drug use is criminalized, public health–oriented interventions remain unevenly developed and largely concentrated in larger urban centres. As a result, people who use drugs often rely on informal, peer-based sources of information, with online drug forums playing a particularly important role in the circulation of user-generated health knowledge and harm reduction practices.

### Prior work

Many studies have addressed health information quality on social media platforms such as YouTube and TikTok. Researchers have noted that the quality and reliability of medical videos vary; most studies have emphasized that, despite their high popularity and number of views, they are generally of low quality, with some bearing signs of disinformation. For example, videos about cancer, vitiligo, diabetes, or hysteroscopy are often incomplete or misleading, and their level of credibility could be assessed as low to moderate [9–13]. Although videos created by medical experts have better substantive ratings, their accessibility and reach are often worse than those created by amateurs, which results in lower engagement [10, 14–16]. The same is true of content shared by non-profit organizations, which is widely commented on but worse in terms of information quality than content shared by commercial entities [12]. Many studies have shown that higher-quality materials do not always reach a wide audience, which may lead to the spread of false beliefs and users making health decisions based on unverified sources [16–20].

Several studies have highlighted key reasons for this pattern. First, user-generated content is typically perceived as more relatable and emotionally engaging, especially when presented in the form of personal experience or storytelling [21, 22]. Second, users tend to trust peers who share similar concerns or identities, even in the absence of formal expertise [23]. Third, platform algorithms on services such as YouTube and TikTok are designed to promote content based on popularity and watch time rather than accuracy, which reinforces the visibility of emotionally charged or sensational posts [24]. These mechanisms contribute to the widespread consumption and influence of health-related misinformation, which often outpaces official recommendations in reach and resonance.

A number of standardized tools have been developed to assess the quality of health information, including DISCERN [10, 11, 13, 14, 25–28], the Global Quality Scale (GQS) [10, 11, 13], PEMAT [11, 13, 26, 29, 30], the JAMA score [31], and others [32]. These instruments are widely used to evaluate traditional medical content, such as websites, brochures, or educational videos. However, scholars have noted that such tools were not designed with the dynamics of social media or online forums in mind, where information is user-generated, conversational, and context-dependent [33]. As a result, current instruments are often ill-suited to evaluating health information shared in these digital environments, which calls for the development of more appropriate, platform-sensitive methods.

Additionally, most research has moved toward assessing YouTube and TikTok video platforms and generally understanding medical information about a given disease (e.g., cancer) [10–13, 16, 26, 28, 31, 32]. While internet forums related to drug use have received increasing scholarly attention, the quality of health-related information shared on these platforms—particularly regarding the effects of psychoactive substances—remains under-researched. An exception is a study by Mukherjee et al. [34] that focused on the analysis of content posted on the health forum Healthboards.com. The researchers created a model for automatically assessing the credibility of medical statements. They examined content related to the side effects of popular drugs such as alprazolam (Xanax), ibuprofen, omeprazole, metformin, levothyroxine, and metronidazole. Although several studies have already analysed data generated by patients online to identify drug side effects [35, 36], the health information quality related to psychoactive substances—particularly illegal ones discussed on user forums—remains under-researched.

### Goals of the study

The present article addresses the criticism of standard tools and moves beyond analysing the aforementioned video services. We propose another information assessment tool, as described in the following sections, and our approach aims to comprehensively assess the content of the selected forum (the Polish-language Hyperreal forum, which is dedicated to drug users). We aim to indicate the scope and health information quality of “medical” posts on Hyperreal and to explore how such content may contribute to user-driven knowledge formation and inform harm reduction approaches on this and similar platforms. We asked the following research questions (RQs):

#### RQ1

What is the scope of drug discussions on Hyperreal in a health-related context, including prevailing topics?

#### RQ2

What is the health information quality of posts related to these topics on Hyperreal?

The analysis was based on identifying the most relevant topics on how substances influence various diseases and the health-related effects of using these substances. Additionally, we determined the quality of information on the forum. It is worth mentioning that this was a multimethod study; for both RQs, we applied different methods: quantitative topic modelling for RQ1 and qualitative expert assessment for RQ2. Despite this, these two methods were strongly connected; we elaborate on this in the “Discussion” section.

This study centres on Hyperreal.info’s discussion forum (“Talk”; https://hyperreal.info/talk/) [37], a prominent Polish-language online platform devoted to discussions about psychoactive substances, mainly illegal ones. The forum constitutes a key component of Hyperreal.info (https://hyperreal.info), the oldest and most extensive Polish portal dedicated to drug-related content. In addition to hosting the forum, the site features educational materials, detailed substance descriptions, legal updates, and cultural commentary. Active since 2005, the “Talk” board is widely recognized as the most established and influential online space of its kind in Poland. As of May 13, 2025, it had 212,498 registered users (at the time of writing, not at the time of data collection). However, the number of actual users may be difficult to determine due to possible account switching or people who have withdrawn from participating but whose profiles remain active.

Hyperreal is an important source of knowledge on psychoactive substances on the Polish internet, and Polish researchers have analysed discourses related to drug use within this community and its role in shaping alternative harm reduction narratives [38]. Czerner et al. [38] demonstrated how Hyperreal functions as a distributed system of knowledge production and negotiation, providing a structured, norm-driven space for sharing experiences, seeking legal advice, and receiving social support. They emphasize the emergence of a parallel epistemic order that legitimizes user-based expertise while fostering cautious, self-regulated patterns of use—key components of grassroots harm reduction. Similarly, previous studies on cannabis-related threads within the same forum have explored how specific narratives are articulated and collectively validated by users, often in ways that challenge dominant public health framings [39, 40]. Taken together, these studies highlight the significance of Hyperreal not only as a site of information exchange but also as an arena of narrative construction and informal governance. It is worth mentioning, though, that although we focused on a specific Polish-language website, our study is intended to yield several general conclusions about the quality of health information in online spaces dedicated to discussions on drugs.

## Methods

The study was informed by prior ethnographic engagement with the Hyperreal forum, conducted as part of a larger research project (see the Funding section). Although this article does not present ethnographic data, the process yielded over 50,000 words of research notes that helped contextualize the dataset and guide analytic choices. Building on this contextual knowledge, the present study employed a multi-method design to explore the quality of health information on an online forum. Specifically, we combined large-scale topic modelling with manual quality expert assessment to provide both macro-level insights and fine-grained evaluations.

### Topic modelling

The aim of the topic modelling analysis was to identify the main themes discussed on the forum and quantify their relative prevalence. For this research, the full content of Hyperreal—1,767,568 posts—was scraped in November 2023. We began the semi-automated content analysis procedure by pre-processing the textual data [41]. This included (1) the removal of platform artifacts (dates of publication at the beginning of the post and functionalities such as the words “quote” and “report” after the date of publication), phrases such as “user [username] said”, “user [username] is inactive and will not respond to this message”, and users’ mottos (i.e., short quotations or adages attached to the end of posts) and (2) the removal of stop words, lemmatization [42], and the replacement of all drug synonyms with a basic form (e.g., “weed”, “ak47”, and “cannabis” were replaced with “marijuana”). A detailed description of the last step can be found in Section AF1 of the Additional file 1. Here, we will mention that the present study includes substances most frequently discussed and traded on the Polish internet, as reported in other publications from our ongoing project [43].

We detected health-related topics using a semi-automated procedure inspired by co-occurrence graph analysis (graphs where nodes represent words and edges connect nodes that co-occur within a specified context) [44, 45] and synonym-based keyword extraction (we used word embedding models to find synonyms of words in the text) [46]. This approach aims to efficiently identify and analyse health-related topics in the data. The method balances speed and accuracy by quickly generating a list of relevant words and allowing human experts to review and select the most useful terms for analysis. First, we used a list of words that appeared in the dataset more than 5000 times (*N* = 2249) and manually selected all health-related words based on the following categories: medicines and supplements (e.g., paracetamol), medical conditions (e.g., hypertension), medical specializations (e.g., cardiologist), medical procedures (e.g., pharmacotherapy), body parts and tissues (e.g., pancreas), processes within organisms (e.g., metabolism), and sources of medical knowledge (e.g., PubMed). The threshold was set to allow for the capture of common words while also keeping the list concise enough for human verification. We selected 240 words in this step. Next, we built vector representations of words (word embeddings, i.e., numerical representations of words). While co-occurrences are limited to words in the same document, word embeddings identify similarities between words based on similar local contexts (shared vector space), which means that they do not have to appear in the same document [47]. In this step, we used the skip-gram approach with 10 words of a slide window to predict the context given the word and capture a broader range of semantic relationships, not only exact synonyms [48]. Next, we created a list of words that were semantically related to our initial list by keeping words with a cosine similarity score of above 0.7. Cosine similarity determines the similarity between two vectors and ranges from − 1 to 1. A cosine similarity of 1 indicates that the vectors are identical, 0 means they are perpendicular (unrelated), and − 1 signifies that they are diametrically opposed. A cosine similarity value of 0.7 indicates a relatively high degree of similarity between two word vectors. This procedure enabled the expansion of the initial list of words using words that were semantically related to them. Finally, the updated list was manually verified based on the previously described criteria. We detected 1611 keywords, including 254 specifically related to the overarching topic of medical treatment (i.e., medical conditions, medical specializations, medical procedures, medicines, and supplements). The full list of 254 keywords used in this study can be found in Section AF2 of Additional file 1.

**Fig. 1:**
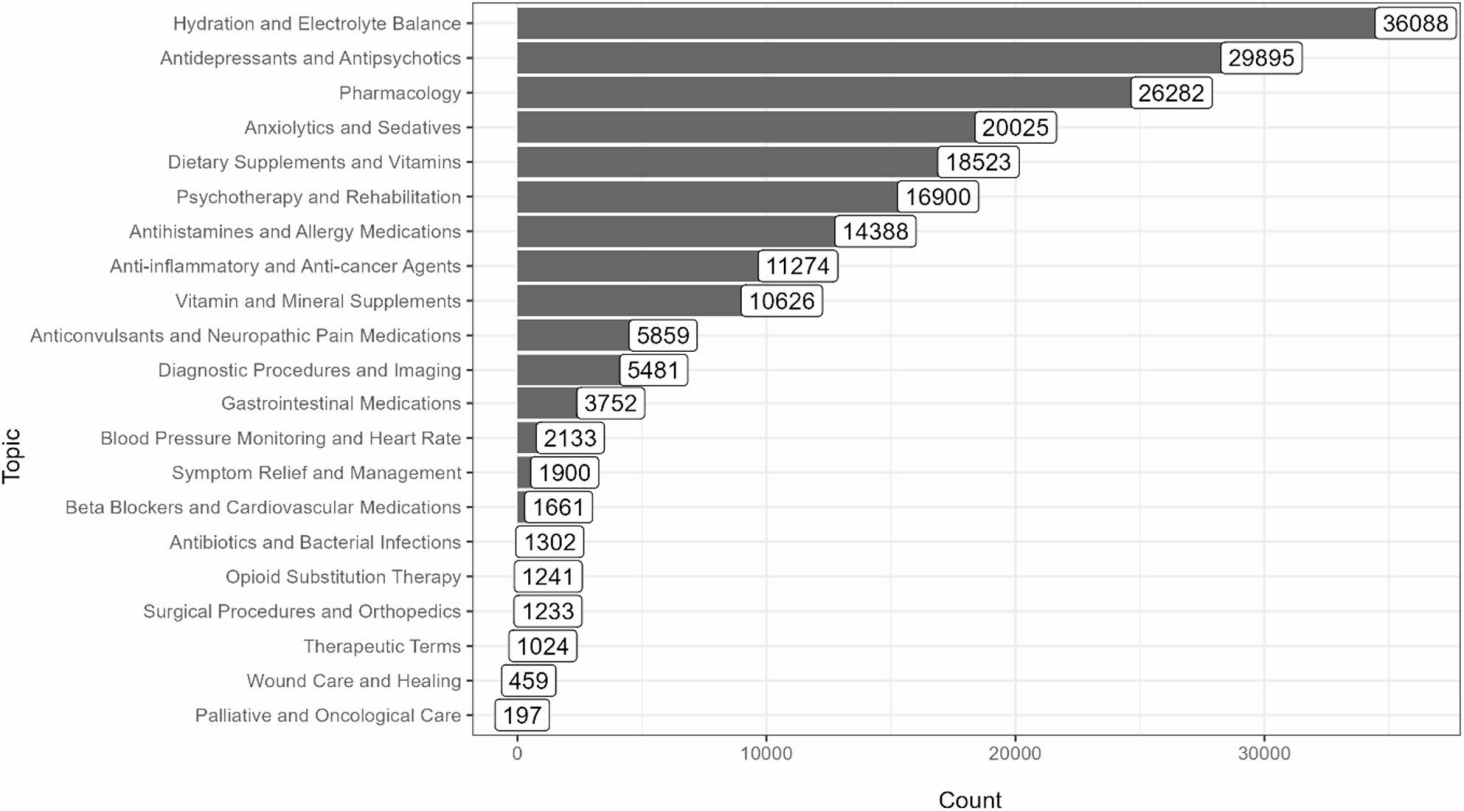
Distribution of subtopics related to documents on drugs and treatment

We narrowed down the textual data to cases in which psychoactive substances were discussed in a medical context and consisted of more than five words. This resulted in a database of 159,145 documents. In the next step, we used previously calculated cosine similarity scores to find all words that were semantically related to keywords in medical treatment documents. Based on this information, we created a network graph with words as nodes and cosine similarity as edges. This allowed us to use the Louvain community detection algorithm [49] and divide words into 21 smaller and more semantically cohesive groups that represented topics (see Fig. 1 in the “Results” section for the distribution).

**Fig. 2:**
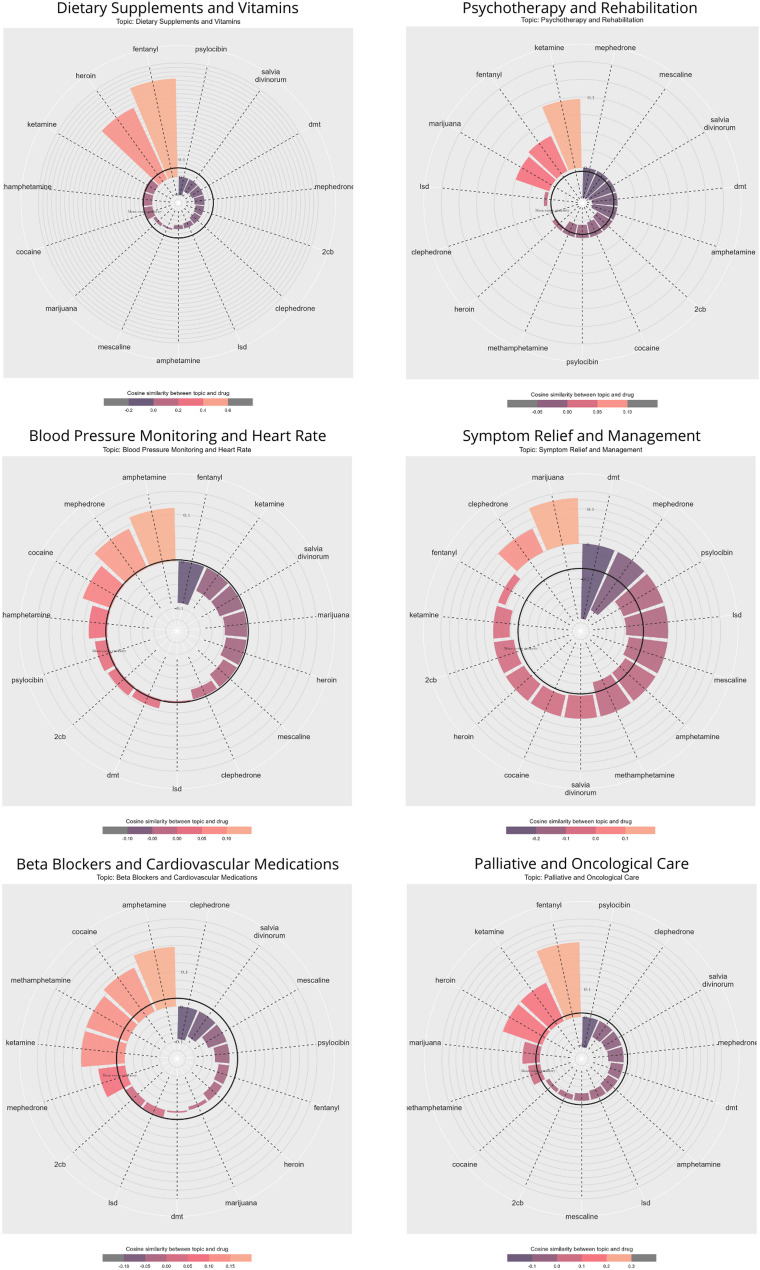
Selected radar plots illustrating semantic relationships between given topics and substances

We used information about keywords for a given topic to calculate the cosine similarity between them and substances. This allowed us to visualize semantic relationships between given topics and drugs and present them on radar plots, which we analyse in the next section (see Fig. 2).

### Expert evaluation

The above dataset of 159,145 posts was also used to draw a random sample of 100 documents for each topic. This resulted in a pool of 2100 randomly selected posts, from which 25 were purposefully chosen. The selection aimed to ensure diversity in terms of the substances discussed and the topics of the posts, such as effects, methods of use, physiological impact, or substance combinations.

These 25 posts were then subjected to expert evaluation by four psychiatrists to assess the quality of health information. Initially, we planned to use one of the previously mentioned tools, such as DISCERN, GQS, PEMAT, JAMA score, or others. However, as mentioned, we decided they were unsuitable for this purpose. Therefore, we developed a questionnaire to assess the quality of the posts, and experts evaluated them in two stages. The first step was to judge the tool itself and submit corrections to it. After all comments were collected and the tool was improved, the experts were asked to assess the 25 posts. The final form of the tool is presented in the next section. We asked each expert to declare their competencies in rating the presented material. All of them indicated that they were competent to make the judgment.

Given the qualitative nature of the expert evaluation, our aim was not to statistically generalize the quality of health information across the entire dataset, but rather to illustrate key patterns and challenges present in user-generated drug-related content. Assessing even 0.5–1% of the 159,145 posts (i.e., hundreds or thousands of entries) would not have been feasible through expert psychiatric review. Moreover, such large-scale expert evaluations are extremely rare in related studies, including those cited in this paper [10, 11, 13, 14, 25–28]. Instead, we followed the qualitative tradition of purposefully selecting diverse examples to capture a spectrum of thematic content. It is also important to emphasize that the selection of posts was not entirely arbitrary. Although the final selection was purposive, it was informed by the earlier topic modelling step, which allowed us to draw posts from each of the 21 identified topics. In this way, topic modelling provided a structured layer of stratification, introducing a level of systematic diversity akin to randomization within each thematic cluster. This multi-step process aimed to ensure that the posts reflected a broad range of issues and substances discussed on the forum. Thus, our approach combined computational topic mapping with qualitative analysis, and we consider the expert insights to be complementary to the broader, data-driven overview.

The four psychiatrists who participated in the expert evaluation were recruited via collaborating institutions, including university-affiliated therapeutic centres and trusted professional networks. These experts had clinical experience in addiction psychiatry and/or psychopharmacology and were familiar with the types of substances and health-related issues discussed in the forum posts. Recruitment was based on the availability and willingness of participants. Although the number of experts may appear limited, it aligns with established practices in qualitative content evaluation and expert judgment studies cited previously [10, 11, 13, 14, 25–28].

### Ethical considerations

The study received ethical clearance from two institutional review boards (see the proper section of the article). Although the Hyperreal forum is publicly accessible and users post under pseudonyms, we recognize that the content may contain sensitive information related to drug use, mental health, and self-treatment practices. We obtained permission from the administrators of the Hyperreal forum to access and extract its content. Before data collection, we also reviewed the site’s robots.txt file, which confirmed that there were no general restrictions on accessing the forum content. Although the file specified a general crawl delay, this served merely as a technical safeguard against server overload.

Additionally, we adopted a cautious approach to minimize potential harm to forum participants. We refrained from disclosing usernames, links, or other identifiable metadata. Example excerpts were translated into English and selectively quoted in the paper with complete anonymization. Additionally, although we initially considered including all 25 full posts in the Additional file, we ultimately decided against this due to ethical concerns about user identifiability and the potential for recontextualization.

Our approach is informed by ongoing debates in internet research ethics, where researchers have highlighted the need to balance public accessibility with participants’ reasonable expectations of privacy, even in open online spaces [50–52]. We view online forums not only as data sources but also as communities that deserve careful and respectful handling in research.

## Results

### Topic distribution analysis

The distribution of topics on Hyperreal suggests some main areas of interest for users who discussed psychoactive substances in a health context. As shown in Fig. 1, the most dominant issues were “hydration and electrolyte balance”, “antidepressants and antipsychotics”, general “pharmacology”, and “dietary supplements and vitamins.” However, their prominence should be interpreted with caution. Aggregating posts at the topic level does not allow us to determine whether this pattern reflects broad user interest or intensified activity by a smaller number of highly active contributors. Nevertheless, regardless of its underlying distribution across users, the visibility of these topics indicates a sustained discursive focus on the effects of substances on basic physiological functions and their interactions with various drugs used in mental health therapy and general pharmacology.

At the other end of the spectrum, topics such as “palliative and oncological care”, “wound care and healing”, and those related to surgical procedures or opioid therapy garnered significantly fewer posts. This may indicate a lower interest among users or fewer people on the forum who struggled with these health problems. Overall, the results suggest that users focused on discussing the direct health effects of drugs and their impact on body balance and mental health.

### Semantic relationship with substances

The next step was to examine the semantic relationships between the given topics and drugs, illustrated by selected radar plots in Fig. 2 (all radar plots are provided in Additional file 1 [AF3]). The results indicate varying degrees of relationships, with certain substances strongly associated with specific health themes. By “associations”, we do not mean co-occurrence at the level of individual words or simple correlations but rather the shared context in which words appeared. For instance, “amphetamine” and “blood pressure” tended to have a higher cosine similarity than “fentanyl” and “blood pressure”, even if these words never appeared together in the exact text. This is because the first pair shares a context with words such as “heart” and “cardiovascular”, whereas the second pair has fewer overlapping contexts. This highlights a key advantage of word embeddings: their ability to uncover relationships based on broader contextual patterns rather than relying solely on direct co-occurrence. Our findings showed the strongest associations in discussions of dietary supplements and vitamins, psychotherapy and rehabilitation, antihistamines, anti-inflammatory and anti-cancer agents, blood pressure, beta blockers, symptom relief and management or opioid substitution therapy, surgical procedures, or palliative and oncological care (all radar plots are provided in Additional file 1, Section AF3).

Certain psychoactive substances were found to have particularly clear associations with specific health topics (for these cases, illustrative radar plots are presented in Fig. 2). For example, ketamine was often discussed in the context of depression or psychotherapy, which may suggest that some users employ it as an alternative treatment for drug-resistant depression. This aligns with research on the antidepressant properties of ketamine, which has been used in therapy [53]. Another example is marijuana, which often appeared in discussions on relieving and managing symptoms. This may indicate that forum users discussed the use of cannabidiol (CBD) for their relaxing and pain-relieving properties and tetrahydrocannabinol (THC) for their well-being. Additionally, marijuana was discussed in the context of alleviating inflammations and its anti-cancer properties, which also mirrors some research, especially on medical marijuana [54]. The connections between topics related to blood pressure and cardiovascular issues and amphetamine also seemed understandable, as psychostimulants, which include amphetamines and novel amphetamine derivatives, are known to affect cardiovascular functions [55]. Thus, users may share information about the effects and risks of these substances. Another example is fentanyl, which was especially discussed in the case of “palliative and oncological care”, as well as “anxiolytics and sedatives” and “dietary supplements and vitamins.” On the one hand, this may suggest that users discussed the potential of fentanyl in the context of managing severe pain. On the other hand, it is also possible that there were discussions on the forum about supplementation for deficiencies resulting from fentanyl use.

Overall, the connections between themes and substances seem to suggest users’ need to discuss self-medication and an experimental approach to managing mental and physical health, which aligns with the “harm reduction from below” paradigm [7, 8, 56]. These findings indicate that users approach drugs as both a source of health challenges and a way to cope with these challenges, which can lead to risky substance use and side effects. Therefore, the next step was to determine the quality of health information on Hyperreal.

### Expert evaluation of health information quality

As mentioned above, a specially developed tool was used to assess the content of the posts. The tool included 13 questions divided into three sections. The first section concerned the reliability of knowledge and compliance with evidence-based medicine, the second focused on safety, and the third was titled “Authenticity and personal experiences.” As indicated, the assessment process involved four psychiatrists who assessed 25 randomly selected posts. Each question could be rated on a scale of 1 to 5, where 1 corresponded to “no”, 5 corresponded to “yes”, and 2 and 4 corresponded to “partially” (for Question 13 only, 2 and 4 corresponded to “moderately”).

For each post, the minimum score for all experts was 13, and the maximum score was 65. The overall mean for all posts was *M* = 35.67, SD = 6.70, which indicates an average quality of health information. Detailed mean scores for individual posts are provided in Section AF4 (Table AF4) of Additional file 1. However, given the aim of this research, we focused on analysing sections and questions from the questionnaire. The mean scores for all individual questions and sections are presented in Table 1. The complete questionnaire is provided in Section AF5 of Additional file 1 in the original Polish and translated into English.

**Table 1:** Quality assessments according to sections and questions

Sections and questions of the questionnaire	Mean	SD
Section 1. The reliability of knowledge and evidence-based medicine	2.79	1.19
Question 1. Is the content consistent with current medical guidelines or evidence-based medicine (speaking colloquially—is the provided information accurate)?	3.55	1.03
Question 2. Does the content consider individual differences in reactions to substances?	2.18	1.02
Question 3. Does the content include information on tolerance and/or synergy between substances?	2.98	1.16
Question 4. Does the content consider the long-term effects of using a given substance?	2.44	1.07
Section 2. Safety	2.52	1.05
Question 5. Does the content include information that can be considered safe for readers?	2.68	0.92
Question 6. Does the content take into account the potential limitations of the provided information?	2.69	1.03
Question 7. Does the content consider potential risks associated with substance use?	2.92	1.03
Question 8. Does the content include information about dosage differences?	1.99	1.02
Question 9. Does the content present safety guidelines for using the substance?	2.33	0.95
Section 3. Authenticity and personal experiences	2.91	1.28
Question 10. Does the content focus on facts?	3.01	1.02
Question 11. Can it be said that the information is not overtly anecdotal and subjective?	3.77	0.93
Question 12. Does the content encourage consultation with specialists?	1.95	1.18
Summary		
Question 13. Assess the overall quality of the content as an information source on substances	3.18	0.99

The answers to the first question show that the content on Hyperreal could be characterized by relatively high compliance with current medical knowledge. Similarly, scores were higher for Question 10, which concerned focusing on facts, and Question 11, which concerned posts not being anecdotal. Therefore, users appeared to try presenting information based on reliable sources and scientific research, even if these were not directly cited. For example, one analysed post scored highly for Question 1: “strong opioids: combining oxycodone with strong opioids that are full agonists or opiates (morphine, heroin, fentanyl) significantly enhances its effects on the central nervous system (CNS) and sedation” (part of the post with unique coding identifier 03_04_07).

The scores from Table 1 above suggest a reliance on credible sources. However, the analysis of the remaining answers indicated significant informational gaps in the content. Although questions about the interaction between substances and potential risks and limitations of information received average ranks, issues such as differences in dosage or guidelines on the safety of substance use were rated much lower. Moreover, all posts ranked poorly when experts evaluated whether these posts encouraged consultation with specialists (“Of course, I’m not a doctor or pharmacist, so relying on my advice may not be advisable” [15_02_19]).

Calculated across all posts and experts, the overall mean for the questions was *M* = 2.74, SD = 1.16, which indicates an average level of health information quality (confirmed by the scores for the last, general Question 13; see Table 1). However, as a whole, the assessment suggests a high potential for harm reduction on forums, which we further discuss in the next section. Additionally, the results from Table 2 show that the expert ratings were similar for given sections, especially those concerning the safety and authenticity of the content. None of the experts scored particularly low or high, as presented in Table AF6 in Section AF6 of the Additional file 1.

**Table 2:** The means and standard deviations of the expert ratings for each section

	Section 1 (Questions 1–4)		Section 2 (Questions 5–9)		Section 3 (Questions 10–12)		Summary (Question 13)	
	Mean	SD	Mean	SD	Mean	SD	Mean	SD
Expert 1	2.89	1.23	2.54	1.09	2.12	1.12	3.08	0.91
Expert 2	2.82	1.02	2.47	0.94	2.16	0.93	3.04	0.84
Expert 3	2.55	1.08	2.43	0.99	2.02	1.06	3.00	1.00
Expert 4	2.89	1.38	2.49	1.15	2.06	1.17	3.60	1.12

## Discussion

### Principal results

The study’s main aim was to determine the scope and quality of health information on the Hyperreal forum, especially regarding its potential for harm reduction. For **RQ1** (i.e., determining the scope of drug discussions on Hyperreal), it can be stated that for some topics indicated, the most discussed substances were those also discussed in medical literature in the context of similar topics. An example is research on the use of ketamine for the treatment of drug-resistant depression, which has indicated its effectiveness as a therapeutic agent [53]. As mentioned, similar research has been conducted on marijuana’s role in relieving pain, inflammation, and cancer therapy [54] or the effects of psychostimulants on circulatory system functions [55].

This linking of topics and substances discussed on the forum to scientific literature indicates that platforms such as Hyperreal are important in the shift from professionalized, top-down narratives towards peer-generated, bottom-up discourses [2, 7]. The forum exemplifies a bottom-up space where users actively seek and exchange information on medically significant topics. These discussions reflect grassroots processes of knowledge construction related to psychoactive substances. As such, understanding the quality of health information on these platforms is essential, particularly in light of their potential to support harm reduction practices. Therefore, the importance of health information quality on online drug platforms, an issue raised in **RQ2** of this study, is growing. Although the two parts of our study— quantitative research on the scope of the medical discussions and a qualitative evaluation of their quality—may initially seem separate, they are strongly intertwined and show the importance of online drug platforms as bottom-up spaces that could play a crucial role in harm reduction.

Expert assessment of health information revealed that the forum posts often referred to facts but lacked key details such as precise dosages, risks related to interactions between substances, or tips for safe use. This indicates a general recognition of the principles of evidence-based medicine among users, but there were significant gaps in their knowledge. Overall, the quality of health information could be considered average.

### Comparison with prior work

The study results are only somewhat consistent with previous analyses of the quality of health information on social media platforms such as YouTube or TikTok, where user-generated content is often popular, but its quality is low [9, 15, 26, 28, 32]. Although we cannot directly compare the results because of the different tools used, unlike these platforms, Hyperreal seems to be characterized by better quality health-related information, especially given the users’ focus on evidence-based content. Users share information related to topics discussed in the medical literature that seems to be factual. However, this does not mean that there is no space for improvement, as we also showed that the content was lacking in some areas.

The present study contributes to the existing literature by highlighting the importance of analysing online forums as a source of information on psychoactive substances. In contrast to previous research that focused on particular diseases, video platforms, or general health forums [9, 15, 26, 28, 32, 34], this study focuses on an online space as an important source of knowledge on drugs.

Although our expert evaluation showed that the overall health information quality of posts on Hyperreal was average, this does not negate the platform’s broader significance. Rather than functioning as repositories of expert-verified knowledge, online drug forums serve as dynamic environments for participatory knowledge-making. Users engage in narrative-building processes—combining experiential accounts, shared practices, and fragments of biomedical discourse—to construct situated understandings of drugs, risks, and harm reduction strategies [3, 4].

This collective meaning-making aligns with what scholars have called “vernacular” expertise or “lay pharmacology” [56, 57], in which users navigate uncertainty by developing and circulating context-sensitive knowledge. The topics we identified—e.g., hydration, antidepressants, symptom management—are not random but reflect sustained attention to the body’s internal regulation and safety. Even if the health information quality is only moderate, the forum’s function as a harm-reductive infrastructure emerges from its role in enabling peer support, reputation-based trust, and ongoing discussion about responsible use [2, 7, 58].

While differences in digital access and digital literacy may shape participation in online drug forums, this study does not aim to profile forum users or make claims about their socio-demographic characteristics. Instead, the analysis focuses on the content and structure of health-related discussions. From this perspective, a further contribution of the study lies in providing large-scale evidence of how thematic structures on drug forums relate to broader discursive patterns of care, self-regulation, and informal health governance. It shows that the harm reduction potential of such spaces lies not only in the quality of content (although it is highly important and a highlight of our study) but also in the performative act of sharing and narrating experiences.

### Limitations

The present study has several limitations. First, it focuses on a single forum (Hyperreal), which does not represent the entire spectrum of online communities related to drug topics, despite being the oldest and most recognizable in Poland. In addition, the analysis only featured posts in Polish, which limits the generalizability of the results to other countries and cultures. In addition, the expert assessment reflects the perspectives of psychiatrists trained within a specific national and clinical context, and evaluations of health information quality may differ across medical cultures and training traditions.

The second limitation concerns the nature of the selected methods. Topic modelling enables the identification of users’ main areas of interest. However, the analysis was conducted at the post level rather than the user level, which precludes assessing how evenly specific topics were distributed across forum participants. The study does not capture contextual factors such as the authors’ motivations or the social impact of their statements. In addition, the topic modelling presented lacked unambiguous measures such as precision, recall, or accuracy, which would enable an objective assessment of the quality of generated topics. Additionally, the expert assessment of health information quality was conducted on a limited and purposefully selected sample of 25 posts. Thus, this may not have fully reflected the diversity of content on the forum, especially since the posts were derived from broader discussions on a given substance. However, while the sample of 25 posts is limited relative to the whole dataset, it is worth reiterating that our goal was not to achieve statistical representativeness in expert evaluation, but to conduct a qualitative analysis aligned with methodological standards in similar studies. Another limitation was that the dynamics of interactions between forum users were not analysed; however, these may be crucial in shaping opinions and making health decisions. Finally, although adapted to the specificity of the research, the assessment tool used requires further validation in different contexts.

## Conclusion

Online drug forums enable users to exchange knowledge. However, they can also support them in managing their health and risks related to psychoactive substances by helping them to make more informed health decisions, despite significant gaps in content. This is particularly important for possible harm reduction strategies and interventions, in which access to reliable information is crucial [2, 3, 7, 8]. The results indicate a need to improve the quality of content available on such platforms. In particular, there is a need to provide more detailed information on dosages, interactions between substances, and safe use, and to persuade users to consult specialists. These suggest potential directions for future research on the role of platform moderation and peer-governance in shaping health information environments. How, and to what extent, such mechanisms could meaningfully improve information quality remains an open empirical question. In addition, health educators and drug policy advocates, public health organizations, and NGOs may engage with online communities more actively, offering validated information and responding to misinformation in ways that respect user autonomy and language.

The present study also points to several directions for future research. Further investigations could move beyond large-scale thematic mapping toward in-depth qualitative content analysis, assessing how peer interactions shape health narratives, how users interpret and apply drug-related knowledge, and how such platforms might be leveraged within broader harm reduction frameworks.

It is also important to extend the analysis to other platforms with different affordances and user demographics, such as Reddit, Telegram, or Discord. These platforms often host drug-related content in more fragmented or transient forms, which may present new challenges for harm reduction. Moreover, focusing on emerging drug trends such as fentanyl or novel synthetic opioids is crucial due to their increasing presence and the high risks associated with misinformation or incomplete knowledge [59]. Future research could contribute to a better understanding of the dynamics of such communities and support the development of more effective educational tools. The present study could also be expanded methodologically, particularly with regard to the selection of posts for qualitative evaluation. Future research might employ semi-automated sampling procedures—such as stratified sampling based on topic prevalence or clustering—to achieve broader and more representative coverage of forum content. In addition, crowdsourcing approaches or larger panels of trained coders could be used to expand the evaluation without compromising consistency.

## Supplementary Information

Below is the link to the electronic supplementary material.

Supplementary Material 1

## Data Availability

The datasets generated and/or analysed during the current study are available in the Zenodo repository, 10.5281/zenodo.10810251.
