# Supplementary material for: The quality and scope of health information on online drug platforms: a topic modelling and expert evaluation study of a Polish-language forum
Source: Harm Reduct J. 2026 Feb 16;23:59. doi: 10.1186/s12954-026-01424-y (PMC13015130; doi:10.1186/s12954-026-01424-y)
Supplement: Supplementary file 1 — Supplementary Material 1 [file 12954_2026_1424_MOESM1_ESM.docx]

**The quality and scope of health information on online drug platforms: A topic modelling and expert evaluation study**

**Additional file**

**AF1—Synonyms of drugs**

Hyperreal users use a number of synonyms for drugs. In the preprocessing stage, we replaced all of these with a formal drug name. This process involves several steps. First, we identified the most common synonyms, based on our knowledge. Second, we used word embeddings and cosine similarity to find which words were synonyms for the words we identified previously. Third, we manually verified the list of cosine similarity-based synonyms. The results are as follows.

**2CB**

2c

2cx

bromo

erox

mft

nexus

różowa kokaina

tootsie

tuci

tusi

venus

**Amphetamine**

amf

amfa

amph

Andrzej

Bennies

benzedryna

biała

białko

Black Beauties

dynks

feta

fetka

fetuczi

fetunia

fuga

fuka

fura

furanie

futro

gipsik

Janusz wajs

krajówa

krajówka

krecha

kreska

Mateusz

mączka

Polfa

Prąd

proszek

psychedryna

speed

spid

szczur

szmata

szmeks

szrot

szuwar

szuwax

ściera

ścierwo

śnieg

teruczyni

tynk

Uppers

wajs

white

wład

władać,

władek,

zima

α-metylofenyloetyloamina

**Cocaine**

koks

koka

kolumbia

koko

kokos

krak

crack

jajo

yayo

śnieg

biała dama

coca-cola

dziadek

charlie

kisielek

ameryka

aminy cucące

białe drożdże

chrzan

C17H21O4N,

**DMT**

4aco/4-Aco

ayahuasca

ayahuasca

changa

czanga

Trypamina

Trypta

yage

yopo

**Fentanyl**

Apache

China Girl

China White

Dance Fever

Duro

Durogesic

fent

Fentanylum

Fentka

Goodfellas

Jackpot

Plaster

Tango & Cash

**Heroin**

Big H

Black Tar

brałn

braun

Braun Sugar

brown

Chiva

cukier

diamorfina

grzanie

gzyms

Hel

Helena

Hell Dust

Helupa

Helupa,

hera

Herman

Horse

Iacetylomorfina

kompot

koszulki

makiwara

mączka

Negra

Smack

soczek

Thunder

zupa

**Ketamine**

cat tranquilizer

cat valium

jet k

keta

kit kat

purple

special k

special la coke

super k

**LSD**

acid

blotter

hoffman

kamyczek

karton

kartonik

kwach

kwadrat

kwas

kwaśny

listek

mellow yellow

papier

papiery

plaster

tektura

window pane

znaczek

**Marijuana**

afgan

ak47

alien cookies

amsterdam mist

aunt mary

baba

babka

baczka

baczki

baka

bakanie

bakłażan

big bud

big devil

bilbo

blant

blue cheese

blueberry

bob

boom

bruce banner

bubble gum

california

california mid lemon cherry

carty inocent

cheese cake

cherry punch

chronic

chrust

chwasty

ciastka

critical

czyścioch

darquiri lime

diesel

do si dos

dope

durban poison

dżamba

dżoint

dżuma

edibles

euforia

f-13

farsz

forest fruit

ganda

gandzia

gangsta

ganja

gelato

gibon

girl scout cookies

glo carts

gorilla glue

gras

grass

grease monkey

green afgan

green crack

green crack

green poison

guerilla gold

haze

herb

himalaya

huana

hulkberry

ice

indo

jack berry

jack herer

jack the ripper

jaranie

jaranko

joint

kana

kif

konopa

kush

kwiatki

lolek

majki

makumba

maria

marihuanina

marjanek

mary j

mary jane

marycha

marysia

maryśka

mataro

matex

mazar

mimosa

mj.

moby dick

moonrock

mota

nepal

no-name

northen lights

orange bud

osiedlówka

palenie

palto

passion

peanutbutter breathe

peyote

piękny liść

pietrucha

pineapple express

poison

pojar

popcorn

*produkty zawierające thc (np. dab, olej rso)* olej, olejek

purple

purple maroc

reefer

royal gorilla

sensi

sherbet

shishka

siano

sieja

siejka

sierściuch

sinsemilla

skręt

skun

skunk

smar

smoke

spliff

strawberry lemon

stuff

susz

szpryca

tangie

think different

topa

topka

trawa

trawka

tropicana glue

typhon

wedding cake

weed

white russian

white shark

white widow

wiadro

wiosna

zacier

ziele

zielona

zielone

zielonka

zielsko

ziółko

zioło

**Mescaline**

Cactus

kaktus

Mesc

meska

pedro

pejotl

peyotl

Peyoto

san pedro

trichocereus bridgesii

trichocereus peruvianus

**Metamphetamine**

Dezoksyefedryna

ice

kostki

kości

meta

meth

meth

metyloamfetamina

N-metyloamfetamina

piko

**Mephedrone**

czwórka

mateusz

mef

mefa

mefistofeles

**Psylocybine**

B+

baluny

Ecuadorian

Golden teacher

Grzybki

Grzyby

grzyby halucynogenne

halucynki

halucypki

halunki

humorki

łasiczka lancetowata

łysak wspaniały

magiczne grzybki

magiczne grzyby

muchomorki

psylki

psylocyby

**Salvia divinorum**

boska szałwia

Maria Pastora

Sally

Salvia

salvia divinorum

SD

ska Pastora

szałwia

szałwia czarownika

**AF2—List of keywords related to medical treatment**

The list of keywords we selected using our semi-supervised approach and used to identify documents mentioning and discussing medical consists of the following:

- samoleczenie - self-treatment

- leczenie - treatment

- lecznic - therapeutic

- leczniczo - therapeutically

- leczony - treated

- leczyć - to treat

- leczący - treating

- wyleczenie - cure

- wyleczony - cured

- wyleczyć - to cure

- przeciwzakrzepowy - anticoagulant

- przeciwgorączkowy - antipyretic

- monar - Monar (Polish charity for addiction treatment)

- monaru - Monar (genitive form)

- przeswietlenie / prześwietlenie - X-ray

- rezonans - MRI

- rtg - X-ray

- tomografia - tomography

- tomografiać - to perform a tomography

- usg - ultrasound

- allertec - Allertec

- amertil - Amertil

- antyalergiczny - anti-allergic

- antyhistamina - antihistamine

- antyhistaminik - antihistamine drug

- antyhistaminowo - antihistaminic (as an adverb)

- antyhistaminowy - antihistaminic

- bilastyna - bilastine

- cetyryzynać - cetirizine-based

- claritiny - Claritine

- difenhydramina - diphenhydramine

- feksofenadyna / feksofenadyny - fexofenadine

- flonidan - Flonidan

- histaminowy - histaminic

- klemastyna / klemastyny - clemastine

- lewocetyryzyna / lewocetyryzynać - levocetirizine / levocetirizine-based

- loratadyna / loratadynać / loratydyna - loratadine / loratadine-based

- przeciwalergiczny - anti-allergic

- przeciwhistaminowy - antihistaminic

- virlix - Virlix

- xyzal - Xyzal

- zyrtec - Zyrtec

- łagodzić - to alleviate

- antybiotyk - antibiotic

- antybiotykowy - antibiotic (as an adjective)

- anksjolitycznie - anxiolytically

- anksjolityczny - anxiolytic

- anksjolityk / anksjolityka - anxiolytic drug

- benzodiazepina / benzodiazepinać / benzodwuazepina - benzodiazepine

- bzd - BZD (abbreviation for benzodiazepines)

- dezypramina - desipramine

- klobazać - clobazam-based

- klometiazol - clomethiazole

- klonazepać - clonazepam-based

- klorazepat - clorazepate

- nefazodon - nefazodone

- przeciwlękowy - anti-anxiety

- nebiwolol - nebivolol

- przeciwnowotworowy - antineoplastic / anti-cancer

- adrenolityk - adrenolytic

- amlodypina - amlodipine

- amlozek - Amlozek

- ciśnieniomierz / cisnieniomierz - blood pressure monitor

- chemioterapia - chemotherapy

- kolonoskop - colonoscope

- kolonoskopia - colonoscopy

- antydepresyjny - antidepressant

- mirtazapina - mirtazapine

- przeciwdepresyjny - antidepressant (as an adjective)

- detoks - detox

- detoksykacja - detoxification

- detoksykacyjny - detoxifying

- detoxi - detox (informal)

- metadon - methadone

- odtrucie - detoxification

- odtruwanie - detoxification process

- odtruwać - to detoxify

- odtruć - to detoxify (perfective)

- odwyk - rehab

- odwykowy - rehabilitative

- elektrowstrząs - electroshock therapy

- gabapentyna / gabapentyny - gabapentin

- karbamazepina - carbamazepine

- lamotrygina - lamotrigine

- pregabalinać - pregabalin-based

- przeciwpadaczkowo - antiepileptically

- przeciwpadaczkowy - antiepileptic

- gastrolog - gastroenterologist

- gastroskop - gastroscope

- gastroskopia - gastroscopy

- gojenie - healing

- nawadnia / nawadniaj - hydrate / hydrate (command)

- nawadnianie - hydration

- nawadniać - to hydrate

- nawodnienie - hydration

- nawodnić - to hydrate (perfective)

- powerad - Powerade

- antybakteryjny - antibacterial

- przeciwbakteryjnie - antibacterial (as an adverb)

- przeciwbakteryjny - antibacterial

- przeciwwirusowy - antiviral

- przeciwzapalnie - anti-inflammatory (adverb)

- przeciwzapalny - anti-inflammatory

- amitryptylina - amitriptyline

- arypiprazole - aripiprazole

- fenotiazyna - phenothiazine

- fluoksetyna - fluoxetine

- fluwoksamina - fluvoxamine

- imipramina - imipramine

- klomipramina - clomipramine

- przeciwpsychotyczny - antipsychotic

- rysperydon - risperidone

- tlpd - TCA (tricyclic antidepressants)

- clemastinum - clemastinum

- metoklopramid - metoclopramide

- prometazyna / prometazynać - promethazine

- przeciwwymiotnie - antiemetic (adverb)

- przeciwwymiotny - antiemetic

- neuroprotekcja - neuroprotection

- neuroprotekcyjny - neuroprotective

- diklofenak - diclofenac

- ketoprofen - ketoprofen

- metamizol - metamizole

- naproksen - naproxen

- przeciwbólowo - analgesically

- przeciwbólowy - analgesic

- uśmierzać - to relieve pain

- uśmierzyć - to relieve pain (perfective)

- paliatywny - palliative

- farmakologiczny - pharmacological

- farmakoterapia - pharmacotherapy

- arypiprazol - aripiprazole

- cbt - CBT (Cognitive Behavioral Therapy)

- psychoterapa / psychoterapia - psychotherapy

- psychoterapeuta - psychotherapist

- psychoterapeuć - psychotherapy-based

- przepłukany - rinsed

- przepłukać - to rinse

- płukanka - rinse

- płukać - to rinse

- antydrgawkowy - anticonvulsant

- miorelaksacyjnie - muscle relaxant (adverb)

- miorelaksacyjny - muscle relaxant (adjective)

- miorelaksant - muscle relaxant

- przeciwdrgawkowo - anticonvulsantly

- przeciwdrgawkowy - anticonvulsant

- espumisan - Espumisan

- ipp - PPI (proton pump inhibitor)

- laktuloza - lactulose

- osłonowy - protective

- pantoprazol - pantoprazole

- przeczyszczająco - laxative (adverb)

- sulfogówno / sulfosyf / sulfosyfa - sulfur-based compound (slang)

- xenny - Xenny

- aakg - AAKG (arginine alpha-ketoglutarate)

- aspargin / aspargina / asparginian / asparginianin - asparagine / aspartate

- b1 / b12 / b6 / b9 - vitamins B1, B12, B6, B9

- bcaa / bcaać / bcay - BCAA (branched-chain amino acids)

- biotyny - biotin

- chela - chelate

- chelat - chelate

- chelatować - to chelate

- chlorella - chlorella

- cynk - zinc

- d3k2 - D3K2 (combination of vitamins D3 and K2)

- elektrolit / elektrolić - electrolyte / to electrolyze

- gastrolit - Gastrolit

- htp - HTP (5-hydroxytryptophan)

- izotoniczny / izotonik / izotonika - isotonic

- k2mk7 - K2MK7 (vitamin K2, MK-7 form)

- karoten - carotene

- kreatyna / kreatynać / kreatyny - creatine

- lecytyny - lecithin

- magnez / magnezem - magnesium

- mineral / minerala / mineralizować / mineraly - mineral / to mineralize

- multiwitamina / multiwitaminać - multivitamin

- niacyny - niacin

- omeg / omega / omega3 / omegać / omegi - omega / omega-3

- orsalit - Orsalit

- potas - potassium

- selen - selenium

- suple / suplement / suplementacja / suplementacjać / suplementować - supplement / supplementation

- tauryna - taurine

- tran - fish oil

- tyrozyna - tyrosine

- vigor - Vigor

- vit / vita - vitamin

- wapnieć / wapno / wapń - lime / calcium

- wit / witamina / witaminka - vitamin

- witaminowy - vitamin (adjective)

- wysokomineralizować / wysokozmineralizować - to highly mineralize

- zmać / zmo - ZMA (zinc and magnesium supplement)

- chirurg - surgeon

- chirurgia - surgery

- chirurgiczny - surgical

- mitynga - meeting (informal)

- monoterapia - monotherapy

- terapeuta / terapeutka / terapeuty - therapist

- terapia / terapiać – therapy

**AF3—Semantic relationships between given topics and substances**

**
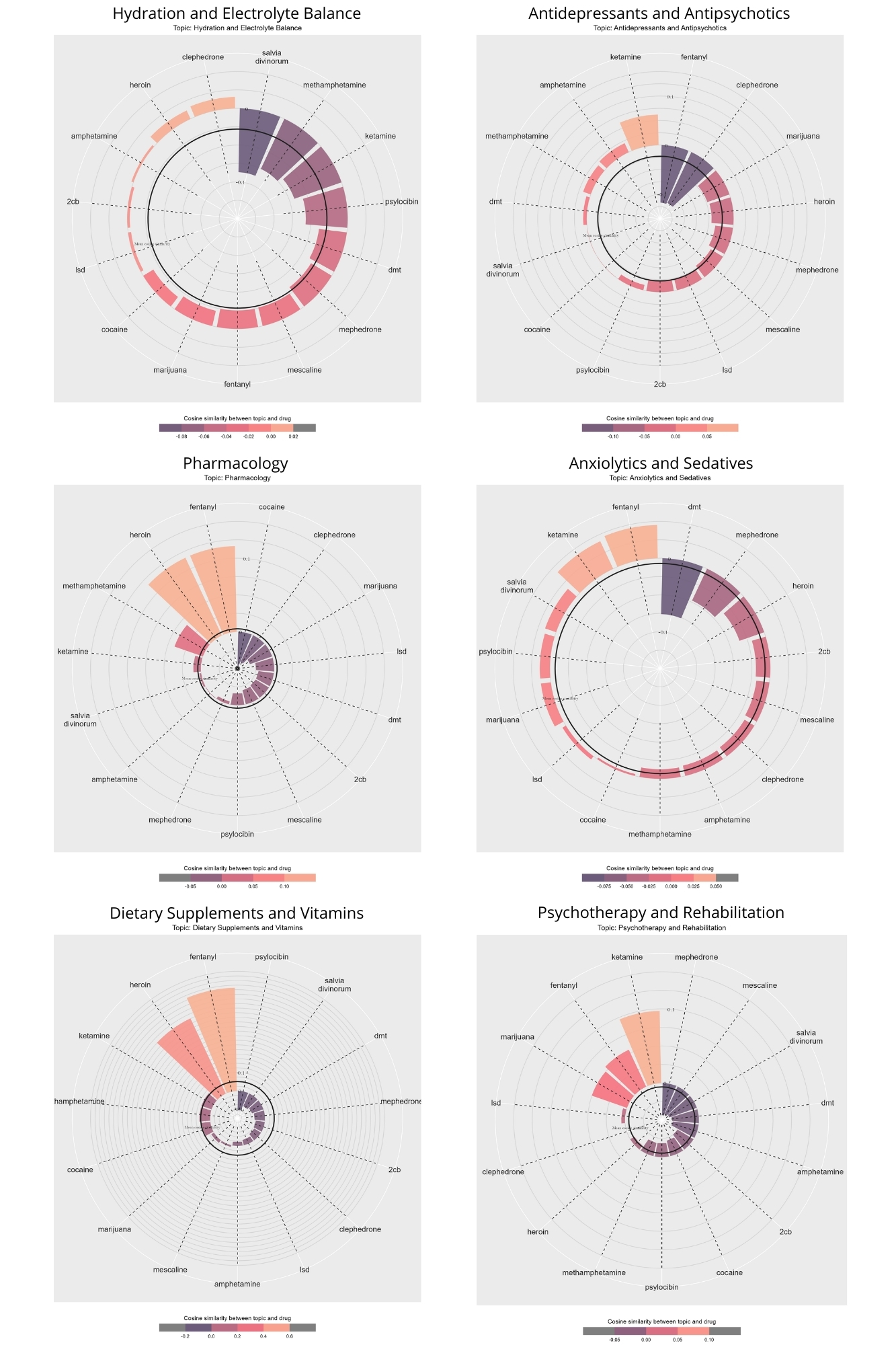
**

**
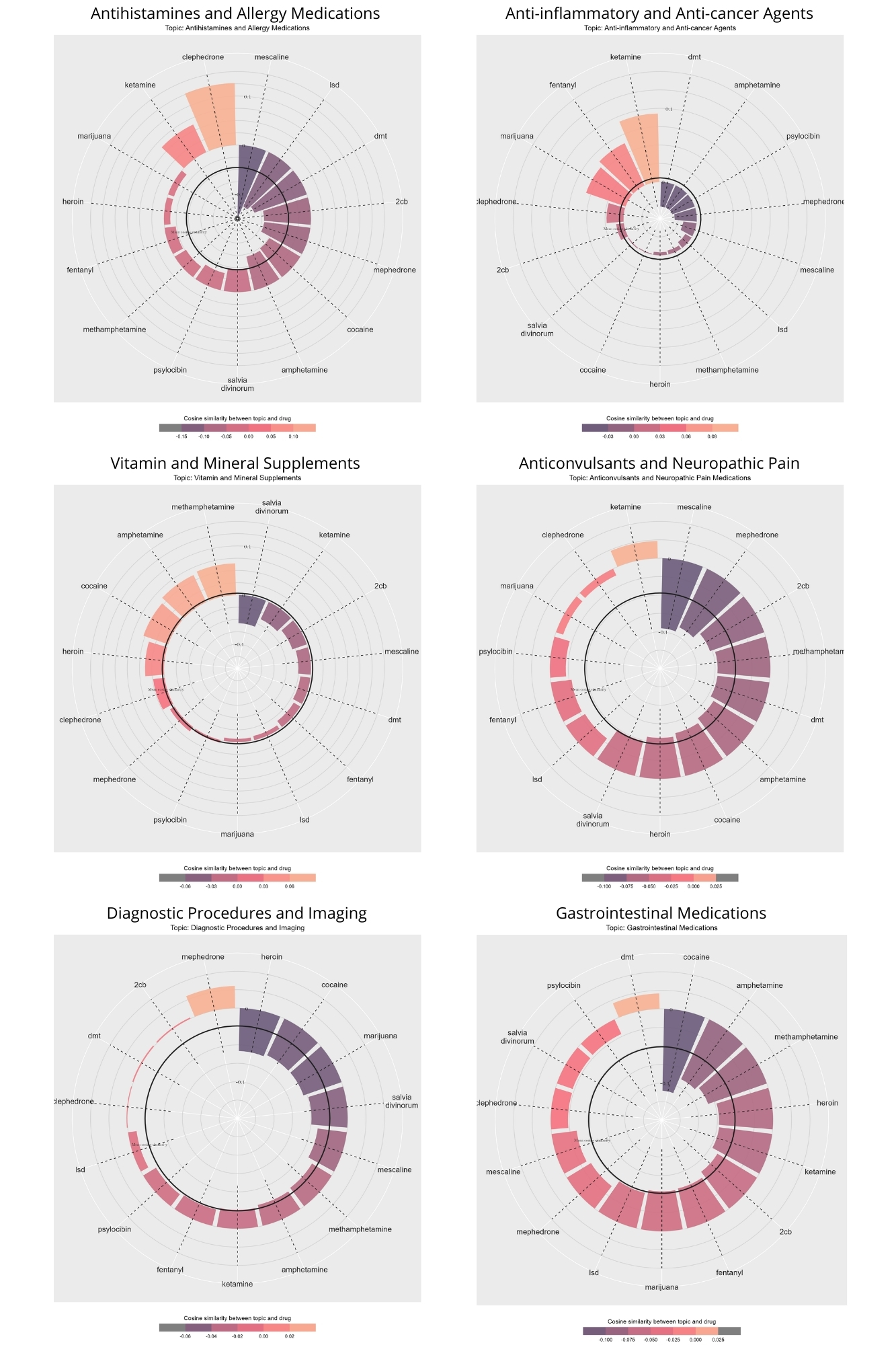
**

**
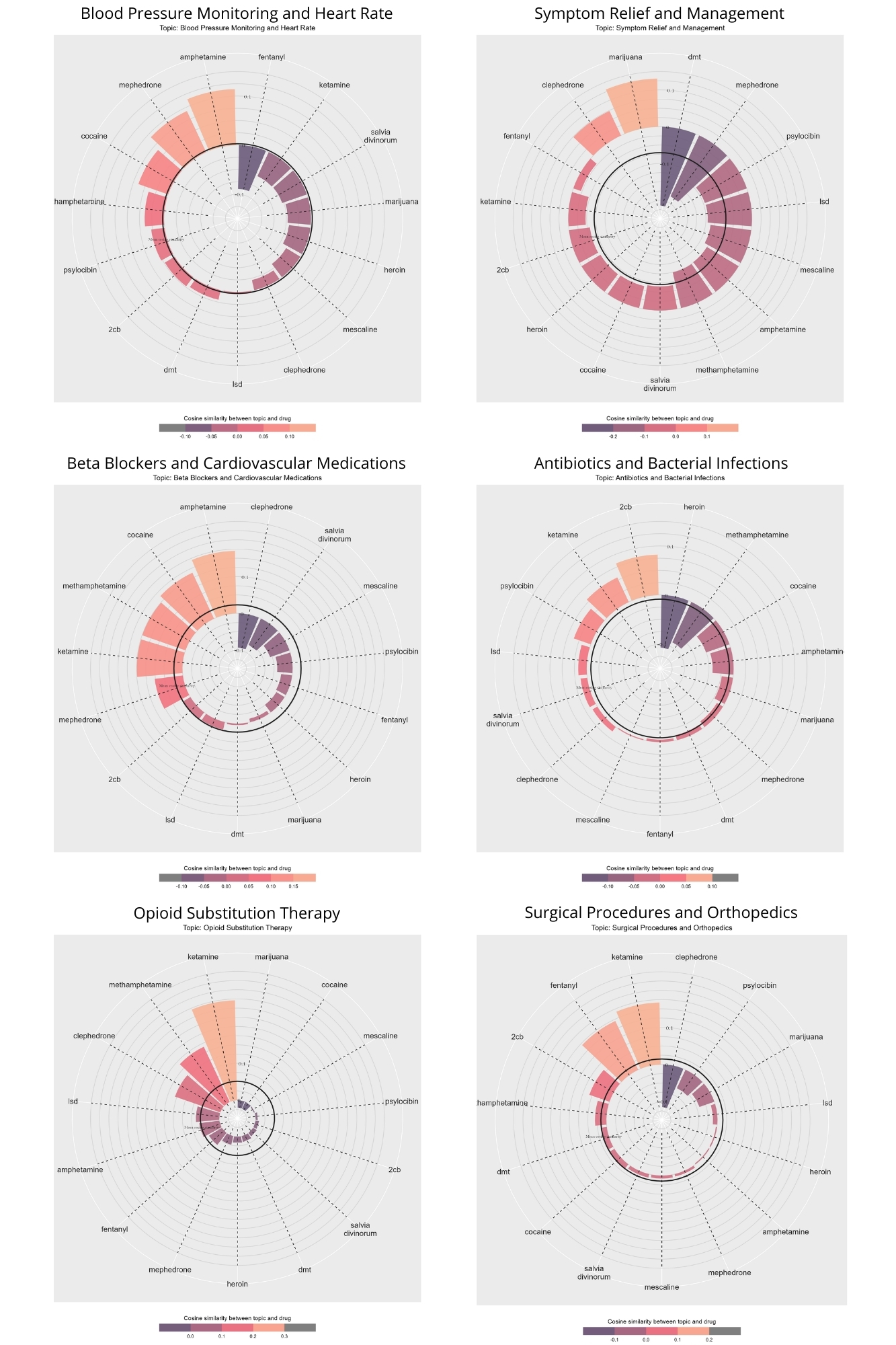
**

**
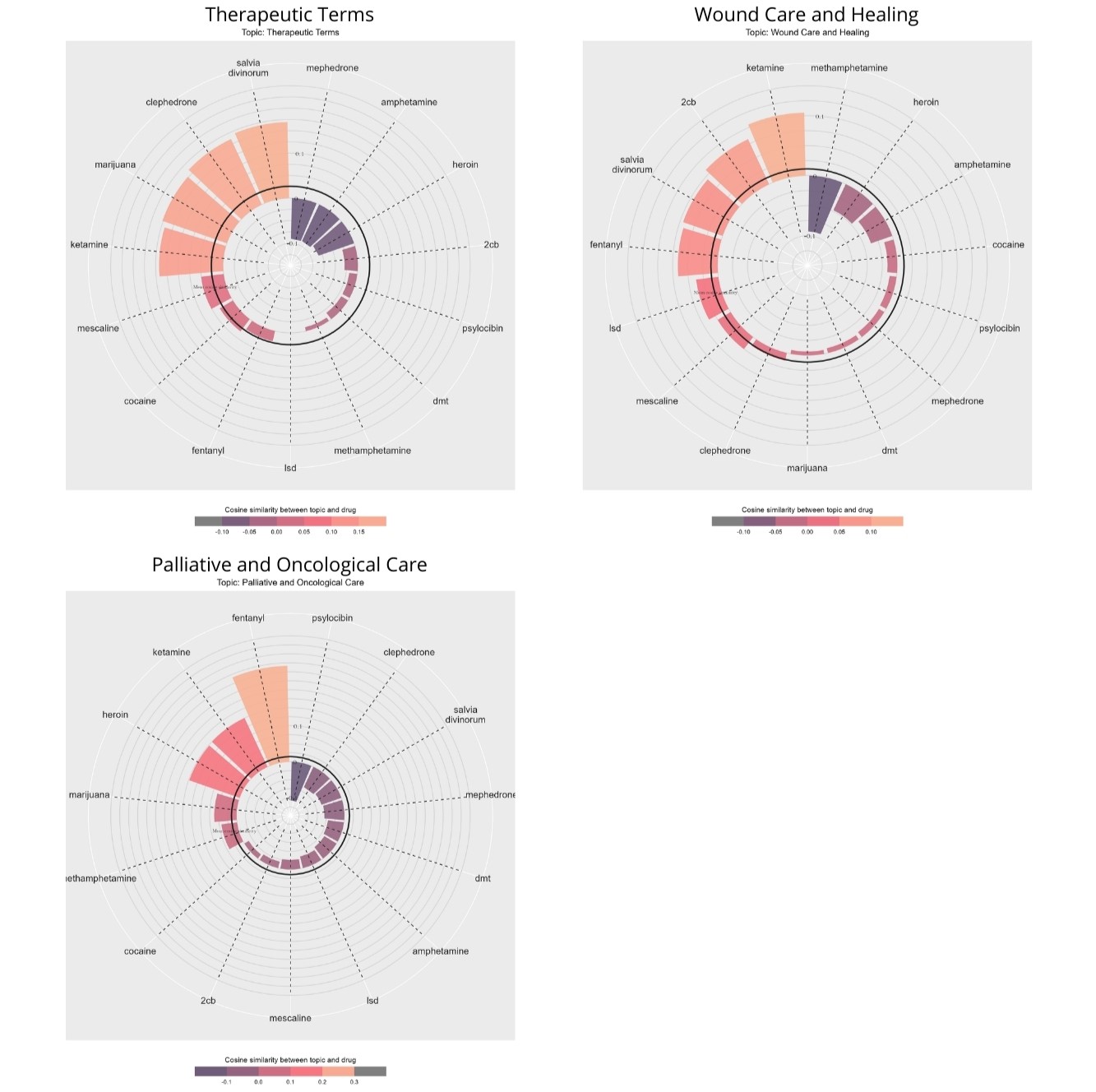
**

**Figure AF3.** Semantic relationships between given topics and substances.

**AF4—Detailed mean scores for individual posts**

**Table AF4**. Quality assessments according to posts.

| **Posts with unique identifiers, means, and *SD*s** | |
| --- | --- |
| **02_01_01**, *M* = 34.25, *SD* = 2.50 | **10_01_14**, *M* = 44.75, *SD* = 2.06 |
| **02_02_02**, *M* = 35.00, *SD* = 2.94 | **12_01_15**, *M* = 30.25, *SD* = 4.57 |
| **02_03_03**, *M* = 37.50, *SD* = 2.38 | **12_02_16**, *M* = 40.25, *SD* = 4.19 |
| **03_01_04**, *M* = 33.75, *SD* = 0.96 | **13_01_17**, *M* = 30.25, *SD* = 0.50 |
| **03_02_05**, *M* = 33.25, *SD* = 2.63 | **15_01_18**, *M* = 45.25, *SD* = 3.30 |
| **03_03_06**, *M* = 40.50, *SD* = 3.87 | **15_02_19**, *M* = 51.00, *SD* = 2.45 |
| **03_04_07**, *M* = 33.75, *SD* = 0.50 | **17_01_20**, *M* = 38.00, *SD* = 3.56 |
| **05_01_08**, *M* = 29.00, *SD* = 1.15 | **17_02_21**, *M* = 44.50, *SD* = 3.87 |
| **07_01_09**, *M* = 31.75, *SD* = 1.71 | **17_03_22**, *M* = 28.50, *SD* = 2.08 |
| **08_01_10**, *M* = 32.25, *SD* = 3.10 | **19_01_23**, *M* = 39.00, *SD* = 1.83 |
| **08_02_11**, *M* = 25.25, *SD* = 1.50 | **21_01_24**, *M* = 32.25, *SD* = 1.26 |
| **09_01_12**, *M* = 38.50, *SD* = 1.29 | **21_02_25**, *M* = 37.50, *SD* = 2.08 |
| **09_02_13**, *M* = 25.50, *SD* = 2.89 |  |

**AF5—Quality of health information questionnaire: a tool used to assess 25 posts (in Polish and translated to English)**

**Kwestionariusz jakości informacji zdrowotnych**

**SEKCJA 1**

Rzetelność wiedzy i *evidence-based medicine* (medycyna oparta na dowodach)

**1. Czy treść jest zgodna z aktualnymi wytycznymi medycznymi lub medycynie opartej na dowodach (*evidence-based medicine*) (mówiąc kolokwialnie – czy podane informacje są prawdziwe)?**

| Nie | Częściowo | | | Tak |
| --- | --- | --- | --- | --- |
| **1** | **2** | **3** | **4** | **5** |

**2. Czy treść zwraca uwagę na indywidualne różnice w reakcjach na substancje?**

| Nie | Częściowo | | | Tak |
| --- | --- | --- | --- | --- |
| **1** | **2** | **3** | **4** | **5** |

**3. Czy w treści jest informacja o tolerancji i/lub synergii między substancjami?**

| Nie | Częściowo | | | Tak |
| --- | --- | --- | --- | --- |
| **1** | **2** | **3** | **4** | **5** |

**4. Czy w treści uwzględnia się długoterminowe skutki użycia danej substancji?**

| Nie | Częściowo | | | Tak |
| --- | --- | --- | --- | --- |
| **1** | **2** | **3** | **4** | **5** |

**SEKCJA 2**

Bezpieczeństwo

**5. Czy treść zawiera informacje, które uznać można za bezpieczne (dla tych, którzy czytają)?**

| Nie | Częściowo | | | Tak |
| --- | --- | --- | --- | --- |
| **1** | **2** | **3** | **4** | **5** |

**6. Czy treść uwzględnia potencjalne ograniczenia podawanych informacji?**

| Nie | Częściowo | | | Tak |
| --- | --- | --- | --- | --- |
| **1** | **2** | **3** | **4** | **5** |

**7. Czy treść uwzględnia potencjalne ryzyka związane z użyciem substancji?**

| Nie | Częściowo | | | Tak |
| --- | --- | --- | --- | --- |
| **1** | **2** | **3** | **4** | **5** |

**8. Czy w treści jest informacja o różnicach w dawkowaniu?**

| Nie | Częściowo | | | Tak |
| --- | --- | --- | --- | --- |
| **1** | **2** | **3** | **4** | **5** |

**9. Czy treść przedstawia zasady bezpieczeństwa podczas używania substancji?**

| Nie | Częściowo | | | Tak |
| --- | --- | --- | --- | --- |
| **1** | **2** | **3** | **4** | **5** |

**SEKCJA 3**

Autentyczność i osobiste doświadczenia

**10. Czy treść koncentruje się na faktach?**

| Nie | Częściowo | | | Tak |
| --- | --- | --- | --- | --- |
| **1** | **2** | **3** | **4** | **5** |

**11. Czy można powiedzieć, że informacje nie mają charakteru wyraźnie anegdotycznego i subiektywnego?**

| Nie | Częściowo | | | Tak |
| --- | --- | --- | --- | --- |
| **1** | **2** | **3** | **4** | **5** |

**12. Czy treść zachęca do konsultacji ze specjalistami?**

| Nie | Częściowo | | | Tak |
| --- | --- | --- | --- | --- |
| **1** | **2** | **3** | **4** | **5** |

**PODSUMOWANIE**

**13. Oceń ogólną jakość treści jako źródła informacji o substancjach.**

| Niska | Umiarkowana | | | Wysoka |
| --- | --- | --- | --- | --- |
| *Rozległe braki* | *Potencjalnie istotne, ale nie poważne braki* | | | *Minimalne braki* |
| **1** | **2** | **3** | **4** | **5** |

**Quality of Health Information Questionnaire**

**SECTION 1**

The reliability of knowledge and evidence-based medicine

**1. Is the content consistent with current medical guidelines or evidence-based medicine (speaking colloquially**—**is the provided information accurate)?**

| No | Partially | | | Yes |
| --- | --- | --- | --- | --- |
| **1** | **2** | **3** | **4** | **5** |

**2. Does the content consider individual differences in reactions to substances?**

| No | Partially | | | Yes |
| --- | --- | --- | --- | --- |
| **1** | **2** | **3** | **4** | **5** |

**3. Does the content include information on tolerance and/or synergy between substances?**

| No | Partially | | | Yes |
| --- | --- | --- | --- | --- |
| **1** | **2** | **3** | **4** | **5** |

**4. Does the content consider the long-term effects of using a given substance?**

| No | Partially | | | Yes |
| --- | --- | --- | --- | --- |
| **1** | **2** | **3** | **4** | **5** |

**SECTION 2**

Safety

**5. Does the content include information that can be considered safe for readers?**

| No | Partially | | | Yes |
| --- | --- | --- | --- | --- |
| **1** | **2** | **3** | **4** | **5** |

**6. Does the content take into account the potential limitations of the provided information?**

| No | Partially | | | Yes |
| --- | --- | --- | --- | --- |
| **1** | **2** | **3** | **4** | **5** |

**7. Does the content consider potential risks associated with substance use?**

| No | Partially | | | Yes |
| --- | --- | --- | --- | --- |
| **1** | **2** | **3** | **4** | **5** |

**8. Does the content include information about dosage differences?**

| No | Partially | | | Yes |
| --- | --- | --- | --- | --- |
| **1** | **2** | **3** | **4** | **5** |

**9. Does the content present safety guidelines for using the substance?**

| No | Partially | | | Yes |
| --- | --- | --- | --- | --- |
| **1** | **2** | **3** | **4** | **5** |

**SECTION 3**

Authenticity and personal experiences

**10. Does the content focus on facts?**

| No | Partially | | | Yes |
| --- | --- | --- | --- | --- |
| **1** | **2** | **3** | **4** | **5** |

**11. Can it be said that the information is not overtly anecdotal and subjective?**

| No | Partially | | | Yes |
| --- | --- | --- | --- | --- |
| **1** | **2** | **3** | **4** | **5** |

**12. Does the content encourage consultation with specialists?**

| No | Partially | | | Yes |
| --- | --- | --- | --- | --- |
| **1** | **2** | **3** | **4** | **5** |

**SUMMARY**

**13. Assess the overall quality of the content as an information source on substances.**

| No | Moderately | | | Yes |
| --- | --- | --- | --- | --- |
| *Extensive deficiencies* | *Potentially significant but not serious deficiencies* | | | *Minimal deficiencies* |
| **1** | **2** | **3** | **4** | **5** |

**AF6—****The means and standard deviations of the expert ratings for each post and each question**

**Table AF6.** The means (*M*) and standard deviations (*SD*) of the expert ratings for each post and each question

|  | **Q1** | | **Q2** | | **Q3** | | **Q4** | | **Q5** | | **Q6** | | **Q7** | | **Q8** | | **Q9** | | **Q10** | | **Q11** | | **Q12** | | **Q13** | |
| --- | --- | --- | --- | --- | --- | --- | --- | --- | --- | --- | --- | --- | --- | --- | --- | --- | --- | --- | --- | --- | --- | --- | --- | --- | --- | --- |
|  | *M* | *SD* | *M* | *SD* | *M* | *SD* | *M* | *SD* | *M* | *SD* | *M* | *SD* | *M* | *SD* | *M* | *SD* | *M* | *SD* | *M* | *SD* | *M* | *SD* | *M* | *SD* | *M* | *SD* |
| **02_01_01** | 3.50 | 0.58 | 2.25 | 0.50 | 2.25 | 0.50 | 2.75 | 0.50 | 3.50 | 0.58 | 2.25 | 0.50 | 1.75 | 0.50 | 2.75 | 0.50 | 1.75 | 0.50 | 2.50 | 0.58 | 3.75 | 0.50 | 1.50 | 0.58 | 3.75 | 0.50 |
| **02_02_02** | 3.50 | 0.58 | 2.25 | 0.50 | 3.50 | 0.58 | 2.75 | 0.50 | 2.75 | 0.50 | 1.25 | 0.50 | 3.00 | 0.00 | 1.50 | 0.58 | 3.75 | 0.50 | 2.25 | 0.50 | 2.75 | 0.50 | 3.00 | 0.00 | 2.75 | 0.50 |
| **02_03_03** | 2.50 | 1.00 | 2.75 | 0.50 | 3.75 | 0.50 | 3.75 | 0.50 | 2.50 | 0.58 | 3.75 | 0.50 | 3.75 | 0.50 | 1.50 | 0.58 | 2.75 | 0.50 | 2.75 | 0.50 | 3.75 | 0.50 | 1.25 | 0.50 | 2.75 | 0.50 |
| **03_01_04** | 4.75 | 0.50 | 1.00 | 0.00 | 1.25 | 0.50 | 2.75 | 0.50 | 3.00 | 0.00 | 2.75 | 0.50 | 2.75 | 0.50 | 1.25 | 0.50 | 1.00 | 0.00 | 3.75 | 0.50 | 5.00 | 0.00 | 1.00 | 0.00 | 3.50 | 0.58 |
| **03_02_05** | 3.75 | 0.50 | 1.25 | 0.50 | 4.25 | 0.50 | 1.25 | 0.50 | 1.00 | 0.00 | 2.00 | 0.00 | 3.50 | 0.58 | 1.25 | 0.50 | 3.25 | 0.50 | 3.25 | 0.50 | 4.75 | 0.50 | 1.00 | 0.00 | 2.75 | 0.50 |
| **03_03_06** | 3.75 | 0.50 | 4.25 | 0.50 | 4.50 | 0.58 | 1.25 | 0.50 | 1.75 | 0.50 | 2.75 | 1.26 | 4.50 | 0.58 | 3.25 | 0.50 | 3.00 | 0.00 | 2.75 | 0.50 | 4.50 | 0.58 | 1.25 | 0.50 | 3.00 | 0.00 |
| **03_04_07** | 4.50 | 0.58 | 1.25 | 0.50 | 4.50 | 0.58 | 1.25 | 0.50 | 1.25 | 0.50 | 2.75 | 0.50 | 3.25 | 0.50 | 1.25 | 0.50 | 1.25 | 0.50 | 4.50 | 0.58 | 3.00 | 0.00 | 1.00 | 0.00 | 4.00 | 0.82 |
| **05_01_08** | 2.75 | 0.50 | 1.25 | 0.50 | 2.25 | 0.50 | 2.75 | 0.50 | 1.25 | 0.50 | 2.25 | 0.50 | 1.25 | 0.50 | 1.25 | 0.50 | 1.00 | 0.00 | 2.75 | 0.50 | 4.00 | 0.00 | 2.75 | 0.50 | 3.50 | 1.00 |
| **07_01_09** | 3.50 | 0.58 | 1.25 | 0.50 | 2.25 | 0.50 | 1.25 | 0.50 | 1.75 | 0.50 | 1.75 | 0.50 | 2.50 | 0.58 | 3.25 | 0.96 | 2.50 | 0.58 | 3.75 | 0.50 | 4.00 | 0.00 | 1.50 | 0.58 | 2.50 | 0.58 |
| **08_01_10** | 3.50 | 0.58 | 1.50 | 0.58 | 1.50 | 0.58 | 1.25 | 0.50 | 3.50 | 0.58 | 2.75 | 0.50 | 2.50 | 0.58 | 1.50 | 0.58 | 1.25 | 0.50 | 3.75 | 0.96 | 4.75 | 0.50 | 1.25 | 0.50 | 3.25 | 0.96 |
| **08_02_11** | 1.00 | 0.00 | 1.50 | 0.58 | 2.00 | 0.00 | 2.25 | 0.50 | 2.50 | 0.58 | 3.00 | 0.00 | 2.50 | 0.58 | 1.25 | 0.50 | 2.75 | 0.50 | 1.50 | 0.58 | 1.25 | 0.50 | 2.75 | 0.96 | 1.00 | 0.00 |
| **09_01_12** | 3.50 | 0.58 | 3.50 | 0.58 | 3.50 | 0.58 | 3.50 | 0.58 | 2.75 | 0.50 | 2.50 | 0.58 | 1.50 | 0.58 | 3.25 | 0.50 | 2.75 | 0.50 | 2.75 | 0.50 | 3.50 | 0.58 | 1.75 | 0.50 | 3.75 | 0.50 |
| **09_02_13** | 2.25 | 0.50 | 1.50 | 0.58 | 2.25 | 0.50 | 1.50 | 0.58 | 1.25 | 0.50 | 2.75 | 0.50 | 2.75 | 0.50 | 1.25 | 0.50 | 1.25 | 0.50 | 1.75 | 0.96 | 3.50 | 0.58 | 2.00 | 0.82 | 1.50 | 0.58 |
| **10_01_14** | 3.50 | 0.58 | 3.50 | 0.58 | 3.50 | 0.58 | 3.75 | 0.50 | 3.75 | 0.50 | 3.50 | 0.58 | 3.75 | 0.50 | 3.75 | 0.50 | 3.75 | 0.50 | 3.25 | 0.50 | 3.25 | 0.50 | 1.75 | 0.50 | 3.75 | 0.50 |
| **12_01_15** | 2.50 | 0.58 | 1.25 | 0.50 | 1.50 | 1.00 | 2.50 | 0.58 | 2.50 | 0.58 | 1.50 | 0.58 | 2.25 | 0.50 | 1.25 | 0.50 | 2.50 | 0.58 | 2.75 | 0.50 | 4.25 | 0.50 | 1.75 | 0.96 | 3.75 | 0.96 |
| **12_02_16** | 4.25 | 0.96 | 1.75 | 0.50 | 3.25 | 0.50 | 3.25 | 0.50 | 4.00 | 0.82 | 4.25 | 0.50 | 2.75 | 0.50 | 2.50 | 0.58 | 3.50 | 0.58 | 2.50 | 0.58 | 3.50 | 0.58 | 1.25 | 0.50 | 3.50 | 0.58 |
| **13_01_17** | 3.50 | 0.58 | 2.00 | 0.00 | 3.75 | 0.50 | 1.75 | 0.50 | 1.75 | 0.96 | 1.50 | 0.58 | 2.50 | 0.58 | 1.75 | 0.50 | 1.75 | 0.50 | 2.50 | 0.58 | 3.50 | 0.58 | 1.25 | 0.50 | 2.75 | 0.96 |
| **15_01_18** | 4.50 | 0.58 | 2.50 | 1.00 | 4.25 | 0.50 | 2.50 | 0.58 | 4.00 | 0.00 | 3.25 | 0.50 | 3.25 | 0.50 | 1.00 | 0.00 | 2.25 | 0.50 | 4.50 | 0.58 | 4.25 | 0.50 | 4.50 | 0.58 | 4.50 | 0.58 |
| **15_02_19** | 4.50 | 0.58 | 3.00 | 0.00 | 4.00 | 0.82 | 4.25 | 0.96 | 4.50 | 0.58 | 3.00 | 0.00 | 3.50 | 0.58 | 3.50 | 0.58 | 3.50 | 0.58 | 4.50 | 0.58 | 4.50 | 0.58 | 3.75 | 0.50 | 4.50 | 0.58 |
| **17_01_20** | 4.00 | 0.82 | 2.75 | 0.50 | 3.50 | 0.58 | 3.25 | 0.50 | 2.75 | 0.50 | 2.75 | 0.50 | 4.50 | 0.58 | 3.00 | 0.00 | 1.50 | 0.58 | 2.25 | 0.50 | 2.50 | 0.58 | 1.25 | 0.50 | 4.00 | 0.82 |
| **17_02_21** | 3.50 | 0.58 | 3.75 | 0.50 | 3.50 | 0.58 | 3.75 | 0.50 | 4.25 | 0.96 | 4.25 | 0.50 | 4.75 | 0.50 | 3.00 | 0.82 | 1.75 | 0.50 | 3.75 | 0.50 | 3.00 | 0.00 | 1.50 | 0.58 | 3.75 | 0.50 |
| **17_03_22** | 4.50 | 0.58 | 1.50 | 0.58 | 1.50 | 0.58 | 1.25 | 0.50 | 3.25 | 0.50 | 1.75 | 0.50 | 1.25 | 0.50 | 1.50 | 0.58 | 1.75 | 0.50 | 2.25 | 1.26 | 3.75 | 0.50 | 1.50 | 0.58 | 2.75 | 0.50 |
| **19_01_23** | 4.25 | 0.50 | 2.75 | 0.50 | 3.25 | 0.50 | 2.50 | 0.58 | 2.75 | 0.50 | 2.75 | 0.50 | 2.75 | 0.50 | 1.25 | 0.50 | 2.50 | 0.58 | 1.75 | 0.96 | 4.50 | 0.58 | 5.00 | 0.00 | 3.00 | 0.00 |
| **21_01_24** | 2.50 | 0.58 | 1.50 | 0.58 | 3.50 | 0.58 | 1.25 | 0.50 | 2.00 | 0.00 | 3.50 | 0.58 | 3.50 | 0.58 | 1.50 | 0.58 | 2.50 | 0.58 | 3.50 | 0.58 | 4.00 | 0.00 | 1.00 | 0.00 | 2.00 | 0.00 |
| **21_02_25** | 4.50 | 0.58 | 2.75 | 0.50 | 1.25 | 0.50 | 2.75 | 0.50 | 2.75 | 0.50 | 2.75 | 0.50 | 2.75 | 0.50 | 1.25 | 0.50 | 2.75 | 0.50 | 3.75 | 0.50 | 4.75 | 0.50 | 2.25 | 0.50 | 3.25 | 0.50 |
